# Preparation of a Co-MXene/CNT Composite for Enhanced Photocatalytic Degradation of Methylene Blue

**DOI:** 10.3390/molecules31101612

**Published:** 2026-05-11

**Authors:** Ming-Zhe Wang, Muhammad Naveed Afridi, Baoji Miao, Kang Hoon Lee, Fengyun Wang, Jinbo Bai, Muhammad Yasir

**Affiliations:** 1 School of Materials Science and Engineering, Henan University of Technology, Zhengzhou 450001, China; ww20022025@163.com (M.-Z.W.); naveed@haut.edu.cn (M.N.A.); 2 Department of Energy and Environmental Engineering, The Catholic University of Korea, Bucheon 14662, Republic of Korea; 3 School of Management, Henan University of Technology, Zhengzhou 450001, China; wfyunxx@163.com; 4 LMPS—Laboratoire de Mécanique Paris-Saclay, Université Paris-Saclay, Centrale Supélec, ENS Paris-Saclay, CNRS, 91190 Gif-sur-Yvette, France; jinbo.bai@ecp.fr; 5 Department of Materials Science & Engineering, Institute of Space Technology, Islamabad 44000, Pakistan; muhammad.yasir@ist.edu.pk

**Keywords:** carbon nanotubes, degradation, methylene blue, MXene, photocatalysis

## Abstract

To overcome the inherent limitations of 2D MXenes in photocatalysis, namely severe nanosheet restacking and rapid charge recombination, this study reports a synergistic dual-modification strategy. By integrating microwave-assisted in situ growth of carbon nanotubes (CNTs) with the hydrothermal incorporation of multivalent cobalt (Co) species, a 3D hierarchical Co-Ti_3_C_2_/CNT composite was successfully fabricated. Structural characterization reveals that the in situ grown CNTs act as robust spatial spacers and conductive highways, effectively preventing Ti_3_C_2_ agglomeration while providing a continuous electron-transfer network. The introduction of Co significantly enriches the surface with redox-active sites and facilitates the formation of an interfacial Schottky junction. Under visible-light irradiation, the optimized Co_10%_-Ti_3_C_2_/CNT composite achieved a superior methylene blue degradation efficiency of 90.3% within 120 min. Mechanistic insights, supported by EPR and electrochemical analyses, confirm that the Schottky barrier at the semiconductor-metal interface acts as a potent electron trap, significantly suppressing e^−^/h^+^ recombination and accelerating surface-mediated radical generation (•OH, •O_2_^−^). This work provides a sophisticated template for designing high-performance, dimensionally stable MXene-based heterostructures for advanced environmental remediation.

## 1. Introduction

Escalating water pollution and the growing scarcity of freshwater resources represent critical bottlenecks for sustainable global development. To mitigate these challenges, the design of low-cost, structurally robust, and visible-light-responsive photocatalysts has emerged as a pivotal strategy for both organic pollutant remediation and green energy conversion [1,2,3]. In recent years, two-dimensional (2D) materials have garnered significant attention in the field of photocatalysis, owing to their distinct layered architectures, high specific surface areas, and accelerated charge carrier dynamics. Since their discovery in 2011, MXenes, distinguished by their metallic conductivity, tunable interlamellar spacing, and rich surface chemistry (e.g., –O, –OH, and –F), have shown immense promise in applications ranging from sensing and energy storage to photoelectrocatalysis. Among these, Ti_3_C_2_T_x_ has emerged as a prototypical MXene, extensively utilized in photocatalytic water splitting, CO_2_ reduction, and wastewater treatment due to its superior electron mobility and versatile interfacial properties [4,5,6].

Despite these advantages, the practical efficacy of pristine Ti_3_C_2_ remains constrained by the rapid recombination of photo-induced charge carriers and a significant loss of active surface area caused by the intrinsic self-restacking and agglomeration of nanosheets [7,8,9]. To address these limitations, various structural engineering and hybridization strategies have been developed to bolster catalytic performance. Notably, the construction of heterojunctions by integrating Ti_3_C_2_ with conventional semiconductors, such as TiO_2_ and ZnO, has proven effective in extending the light-harvesting range and facilitating efficient charge carrier separation kinetics [10,11,12]. Furthermore, utilizing Ti_3_C_2_ as a co-catalyst combined with metal sulfides (e.g., CdS) has yielded substantial enhancements in hydrogen evolution rates under visible-light irradiation [13,14].

Beyond hybridization with inorganic semiconductors, the integration of carbonaceous nanomaterials, such as one-dimensional (1D) CNTs and 2D graphene, has emerged as a robust strategy to enhance charge carrier mobility and structural integrity [15,16]. These carbon-based materials possess exceptional electrical conductivity and abundant interfacial active sites; they act as conductive bridges, facilitating the rapid separation and migration of photogenerated carriers while simultaneously constructing a three-dimensional (3D) hierarchical framework that mitigates the interlayer restacking effects of MXene. In other 2D systems (e.g., g-Ti_3_C_2_, MoS_2_), such composite strategies have demonstrated significantly improved photocatalytic efficiencies [17,18,19].

Furthermore, transition metal incorporation and surface functional group modification offer additional pathways to tune the electronic band structure and interfacial redox activity of MXenes [20,21,22]. For example, nitrogen-doped Ti_3_C_2_ MXene has been shown to substantially enhance organic dye degradation [23], demonstrating a synergistic mechanism between carrier transport and surface reaction activity. Transition metals such as cobalt (Co), characterized by their rich electronic structures and excellent redox activity, can be incorporated into photocatalytic systems to construct interfacial energy levels, thereby optimizing charge carrier separation [24,25,26].

Although several research works have explored MXene-based composite photocatalysts [27,28,29], significant challenges persist—particularly regarding the intricate interfacial engineering of these structures. Moreover, the fundamental mechanisms governing the suppression of charge-carrier recombination remain largely unclarified. Consequently, the development of 3D conductive networks that synergistically combine high specific surface areas with robust structural stability remains a key objective [30,31,32].

Conventional approaches for integrating Ti_3_C_2_ with CNTs have encompassed chemical vapor deposition, laser ablation, electrochemical deposition, and microwave irradiation [33,34]. Furthermore, these binary systems primarily focus on accelerating electron migration, while the rapid recombination of photo-generated holes on the MXene surface remains a critical bottleneck. To distinguish it from previously reported MXene/CNT catalysts, we developed a unique two-step synergistic engineering strategy: microwave-assisted ultrafast in situ growth coupled with site-specific hydrothermal Co incorporation. The specific novelty of this work resides in three aspects: First, unlike traditional CVD or hydrothermal growth, the microwave irradiation triggers localized high-energy plasma effects, driving the in situ nucleated growth of CNTs directly from the Ti_3_C_2_ surface. This establishes a robust, chemically bonded 3D ‘pillared-layer’ architecture that inherently prevents MXene restacking even under rigorous stirring. Second, the strategic introduction of Co ions does not merely act as a dopant but serves as a redox mediator that optimizes the electronic band structure. Third, and most importantly, this work achieves a dual-carrier management mechanism: the in situ grown CNTs serve as ‘high-speed electron freeways’ while the Co-sites act as ‘hole-accumulators’. This synergistic integration addresses the limitations of binary MXene/CNT systems, where hole-initiated reactions are often neglected, thereby leading to a significant leap in photocatalytic efficiency and structural longevity.

## 2. Results and Discussion

### 2.1. Structural Analysis

**Figure 1:**
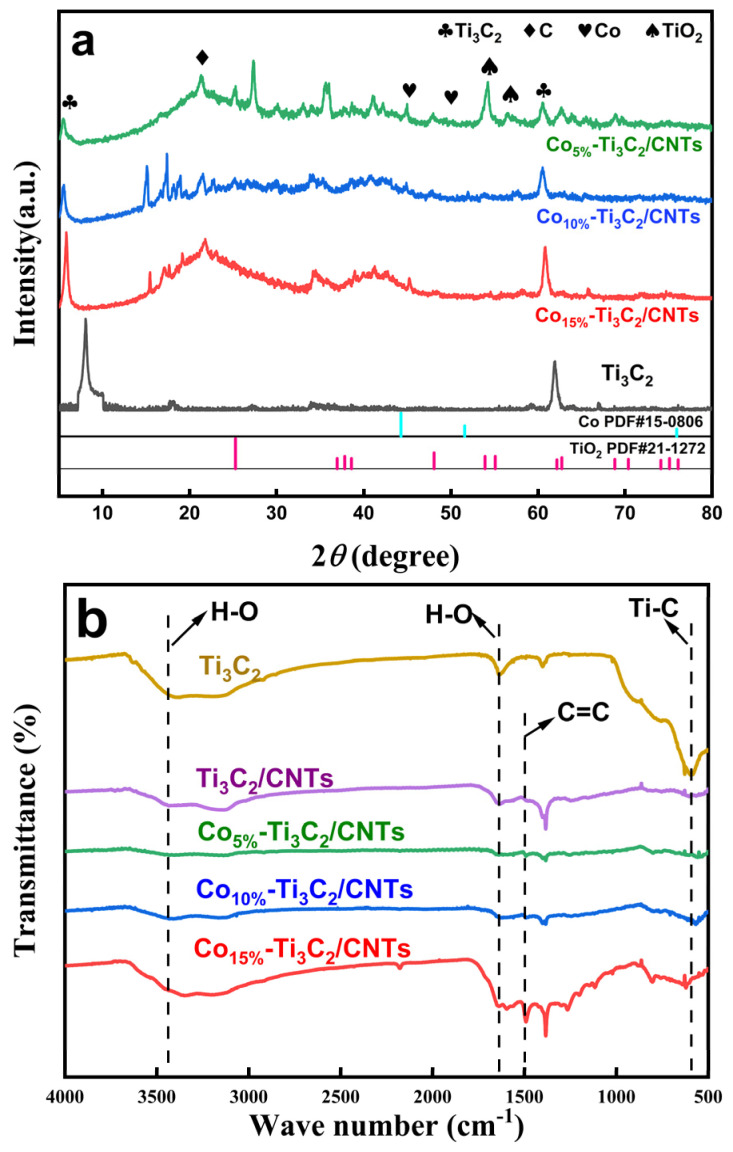
(**a**) XRD patterns and (**b**) FTIR spectra of Ti_3_C_2_ and the Co-Ti_3_C_2_/CNT composite.

The XRD patterns (Figure 1a) illustrate that following LiF/HCl etching, the characteristic (002) and (110) reflections of Ti_3_C_2_ MXene appear, confirming the successful removal of Al atoms from the Ti_3_AlC_2_ MAX phase [35]. Upon the formation of the Co-doped composite, both the (002) and (110) diffraction peaks undergo a noticeable shift toward lower 2θ angles. According to Bragg’s law, this shift indicates an expansion of the interlayer d-spacing, which signifies reduced restacking and enhanced exfoliation of the Ti_3_C_2_ nanosheets. This structural evolution is attributed to the in situ growth and intercalation of CNTs, which act as nanopillars to prevent the van der Waals-driven re-aggregation of the MXene layers. Furthermore, the diffraction peaks at approximately 44° and 51° are indexed to the (111) and (200) planes of metallic Co (JCPDS No. 15-0806), while the reflection at ~26° corresponds to the (002) plane of graphitic carbon (C) from the CNTs. During microwave irradiation, the Co species serve a dual role: catalyzing the carbon source into CNTs while being simultaneously reduced to metallic Co nanoparticles [36]. A minor TiO_2_ phase is also detected, likely resulting from the localized high-temperature environment during microwave treatment, causing partial surface oxidation of the Ti_3_C_2_ MXene.

The FT-IR spectra (Figure 1b) further elucidate the surface chemistry. Pristine Ti_3_C_2_ exhibits a broad, intense absorption band at 3440 cm^−1^, corresponding to the stretching vibrations of surface hydroxyl (–OH) groups [37,38]. Following the integration of CNTs and Co doping, the intensity of this band diminishes, suggesting that the hydroxyl groups are either partially substituted or involved in interfacial coordination with the Co sites. While the composite maintains the characteristic Ti–O and Ti–C skeletal vibrations, high Co loadings induce new vibrational features in the 1000–1600 cm^−1^ region. These emerging peaks likely indicate the formation of strong chemical bonds between the Co nanoparticles and the Ti_3_C_2_/CNT support, or a significant redistribution of surface functional groups caused by the hydrothermal and annealing processes.

### 2.2. Morphological Characterization

**Figure 2:**
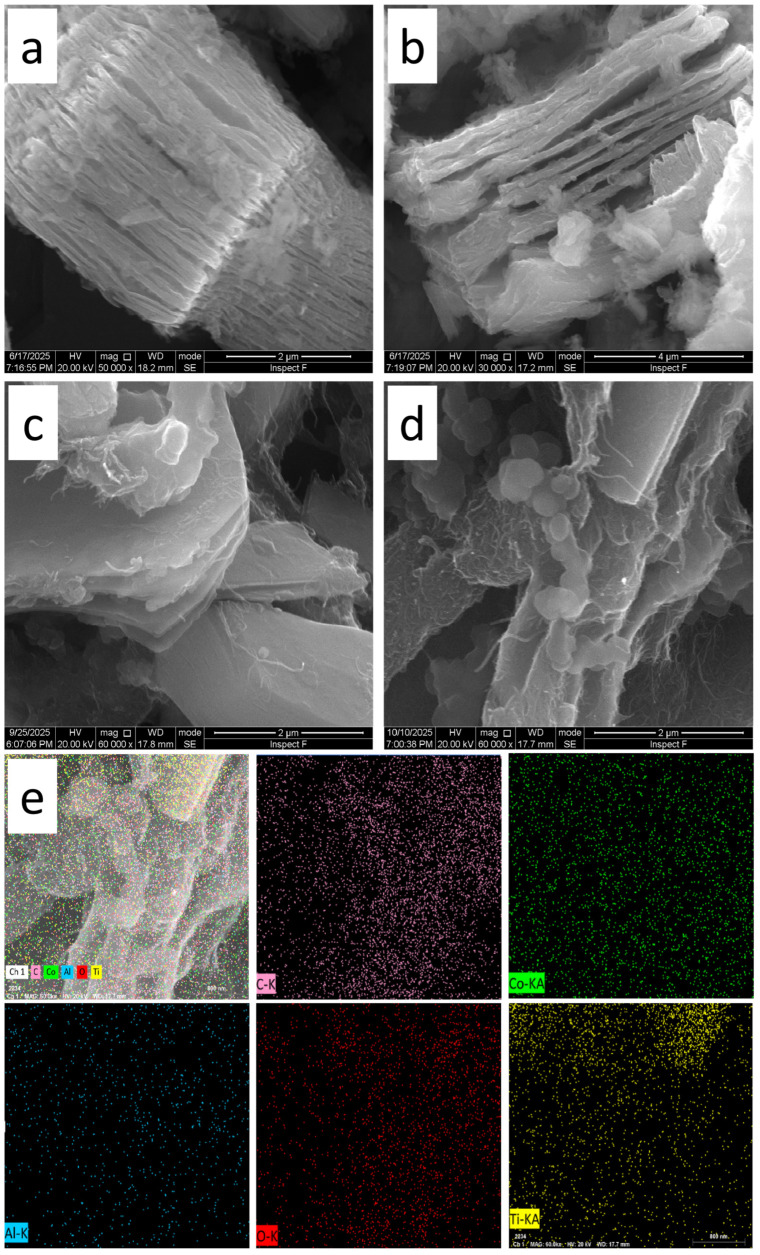
SEM images of (**a**) Ti_3_C_2_; (**b**) Ti_3_C_2_/CNTs; (**c**) and (**d**) Co_10%_-Ti_3_C_2_/CNT composite; (**e**) elemental mapping of the Co_10%_-Ti_3_C_2_/CNT composite.

The morphological evolution of the composites was further elucidated via SEM and elemental mapping (Figure 2). As illustrated in Figure 2a, the LiF/HCl-etched Ti_3_C_2_ exhibits a well-defined, accordion-like layered architecture, which provides an ideal platform for the subsequent in situ growth of CNTs. Following the initial round of microwave irradiation (Figure 2b), the primary 2D layered framework remains largely intact, with only sparse, incipient curved features emerging on the nanosheet surfaces, indicating a nucleating stage of CNT growth. However, upon the second round of microwave treatment with an additional carbon source and catalyst (Figure 2c,d), a significant transformation occurs: a dense, interwoven network of CNTs becomes clearly visible. These CNTs are not merely surface-adsorbed but appear to originate directly from the Ti_3_C_2_ MXene templates and effectively intercalate between the layers [39]. This hierarchical 3D configuration is pivotal for preventing MXene restacking and maximizing the exposure of active sites. The corresponding EDS elemental mapping (Figure 2e) confirms a highly uniform distribution of C, Ti, and Co throughout the composite, highlighting the successful and homogeneous integration of the metal species and the carbon network within the MXene matrix.

**Figure 3:**
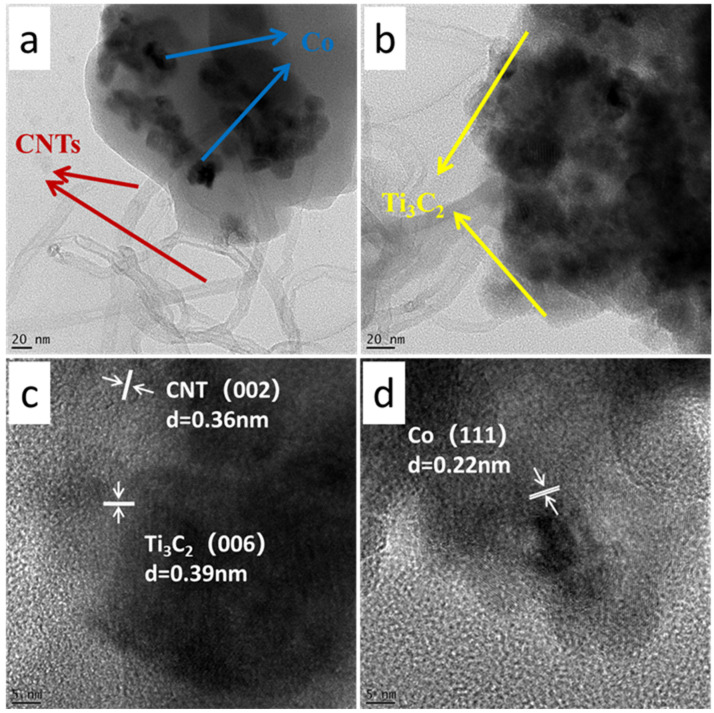
(**a**,**b**) TEM images; (**c**,**d**) HR-TEM analysis of the Co_10%_-Ti_3_C_2_/CNT composite.

To further elucidate the microstructure and interfacial coupling of the Co-Ti_3_C_2_/CNT composite, TEM and high-resolution TEM (HR-TEM) analyses were performed (Figure 3). The TEM images in Figure 3a,b confirm that Co ions are predominantly attached to the Ti_3_C_2_ nanosheets. It is clearly observed that the CNTs originate from and extend outward from the Ti_3_C_2_ templates, effectively creating a cross-linked 3D conductive network. The HR-TEM images (Figure 3c,d) provide definitive evidence of the crystalline phases and their coherent interfaces. Lattice fringes with interplanar spacings of 0.36 nm, 0.39 nm, and 0.22 nm are clearly determined, corresponding to the (002) plane of graphitic CNTs, the (006) plane of Ti_3_C_2_ MXene, and the (111) plane of metallic Co, respectively [40,41]. The close proximity and overlapping of these lattice fringes suggest the formation of a robust heterostructure, which is influential in facilitating the rapid spatial separation and migration of photogenerated charge carriers across the multicomponent interface.

### 2.3. XPS Analysis

**Figure 4:**
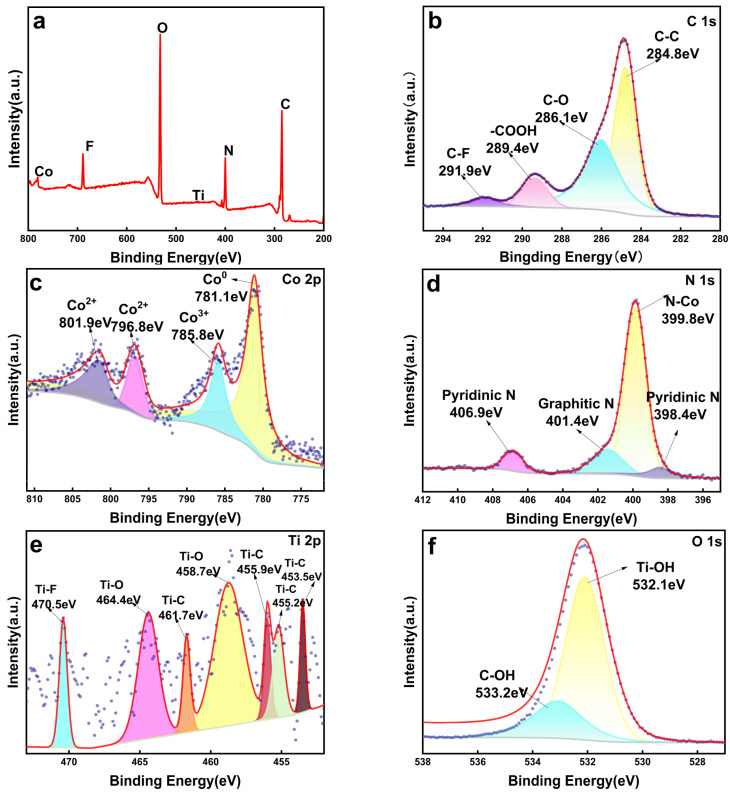
XPS spectra of the Co_10%_-Ti_3_C_2_/CNT composite: (**a**) survey scan; (**b**) C 1s; (**c**) Co 2p; (**d**) N 1s; (**e**) Ti 2p; (**f**) O 1s. The purple dots are the original data points; the red line is the fitted curve; the gray line is the baseline.

XPS was employed to elucidate the surface elemental compositions, chemical valence states, and interfacial electronic interactions within the Co-Ti_3_C_2_/CNT composite. The survey spectrum (Figure 4a) confirms the coexistence of Ti, C, Co, O, N, and F within the hybrid structure. The high-resolution C1s spectrum (Figure 4b) is deconvoluted into four distinct peaks at 284.8, 286.1, 289.4, and 291.9 eV, corresponding to C–C, C–O, C=O/–COOH, and C–F bonds, respectively. The presence of C–F features suggests partial fluorination of the carbon framework, likely originating from the LiF/HCl etching residual. In the Co2p region (Figure 4c), the peaks at 781.1, 785.8, 786.8, and 801.9 eV are attributed to metallic Co^0^ and the cationic species Co^3+^ and Co^2+^. The XPS results show that Co mainly exists in the forms of Co^2+^ and Co^3+^, and no large area of independent oxide phase was found by TEM. This indicates that the cobalt species are highly dispersed and anchored on the surface defects and functional group sites of MXene/CNTs through the hydrothermal process, rather than a simple physical mixture. The identification of Co^0^ confirms the successful reduction of the cobalt precursor into metallic nanoparticles during the microwave process. The coexistence of oxidized species (Co^3+^/Co^2+^) is attributed to the partial surface oxidation of the metallic cores upon atmospheric exposure. This mixed-valence configuration is highly advantageous; the metallic Co^0^ core forms a Schottky junction that acts as an electron sink to trap photogenerated carriers, while the surface Co^3+^/Co^2+^ redox couples facilitate interfacial charge transfer and surface catalytic kinetics. This synergy between the multivalent cobalt species and the conductive Ti_3_C_2_/CNT network significantly enhances photocatalytic efficiency.

The N1s spectrum (Figure 4d) reveals peaks at 399.8, 401.4, and 406.9 eV, indexed to N–Co coordination, graphitic N, and pyridinic N, respectively. The formation of N–Co bonds indicates strong chemical anchoring of the cobalt species onto the nitrogen-doped carbon matrix. The Ti 2p spectrum (Figure 4e) displays characteristic peaks corresponding to Ti–C, Ti–O, and Ti–F bonds. Specifically, the identification of the Ti–C bonds (2p_3/2_ at ~455.2 eV) ascertains that the Ti_3_C_2_ MXene lattice maintains its structural integrity, effectively withstanding the high-temperature microwave treatment during CNT growth. The Ti–O signals (458.7 and 464.4 eV) signify the presence of surface TiO_2_, which serves as nucleation sites for the cobalt species [42,43]. This intimate interfacial contact, further evidenced by the O1s spectrum (Figure 4f) containing Ti–OH and C–OH features (532.1 and 533.2 eV), promotes efficient charge flux from the conductive Ti_3_C_2_/CNT network to the cobalt active centers, thereby significantly supporting photocatalytic performance [43,44,45].

### 2.4. BET Analysis

**Figure 5:**
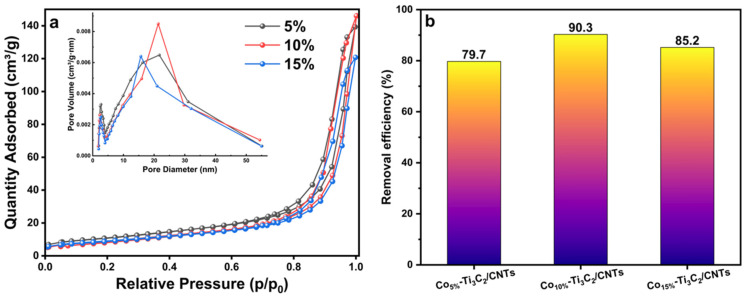
(**a**) N_2_ adsorption–desorption isotherms and pore size distribution curve (inset) of the Co_10%_-Ti_3_C_2_/CNT composite and (**b**) preliminary degradation experiment.

The N_2_ adsorption/desorption isotherms and corresponding pore size distributions of the synthesized composites are shown in Figure 5a. All samples exhibit characteristic Type IV isotherms with H3-type hysteresis loops, signifying a predominantly mesoporous structure characterized by slit-like pores. This morphology is consistent with the layered structure of the MXene and the interstitial spaces created by the interwoven CNT network. The pore size distribution curves (Figure 5, inset) reveal a primary distribution range of 2–25 nm, further confirming the mesoporous nature of the hybrids. The specific surface areas determined using the BET are presented in Table 1. Notably, the Co_10%_-Ti_3_C_2_/CNT composite has a specific surface area of 39.4531 m^2^/g, which is larger than that of Co_5%_-Ti_3_C_2_/CNTs (33.1902 m^2^/g) and Co_15%_-Ti_3_C_2_/CNTs (32.5727 m^2^/g). The superior surface area of the 10 wt% sample suggests an optimal balance between CNT growth and cobalt loading, which effectively inhibits MXene restacking. Generally, a heightened specific surface area provides a greater density of exposed active sites and facilitates the rapid diffusion and adsorption of organic molecules, thereby supporting the overall photocatalytic degradation efficiency.

### 2.5. Preliminary Degradation Experiment

To evaluate the influence of Co loading on the photocatalytic performance of the Co-Ti_3_C_2_/CNT composites, a series of experiments were conducted with varying Co mass percentages (5%, 10%, and 15%). As illustrated in Figure 5b, the photocatalytic degradation efficiency improved significantly with the initial increase in Co loading. Specifically, the 5% Co-loaded sample achieved a degradation efficiency of 79.7%, whereas the 10% composite exhibited the highest performance, reaching a removal efficiency of 90.3%.

This enhanced activity is attributed to the synergistic effect between the Co species and the Ti_3_C_2_/CNT framework; an optimal loading (10%) provides abundant active sites and facilitates the separation of photo-generated electron-hole pairs. However, increasing the Co loading to 15% resulted in a decline in efficiency to 85.2%. This decrease is likely due to the partial aggregation of excess cobalt species, which may shield light absorption or act as recombination centers for charge carriers. Consequently, the 10% Co-Ti_3_C_2_/CNT composite represents the optimal architecture for maximizing active site utilization and charge transfer efficiency.

### 2.6. Photocatalytic Electrochemical Characterization

**Figure 6:**
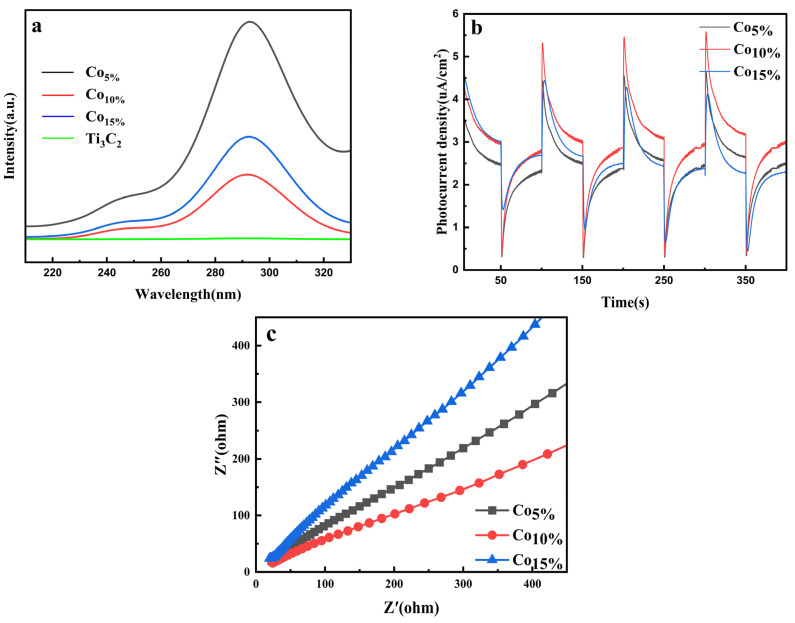
(**a**) Photoluminescence (PL) spectra; (**b**) transient photocurrent response curves; (**c**) electrochemical impedance spectroscopy (EIS) Nyquist plots of the synthesized composites.

The charge separation efficiencies of the synthesized composites were systematically evaluated using photoluminescence (PL) spectroscopy and electrochemical techniques. As shown in Figure 6a, with an excitation wavelength of 200 nm, all samples exhibited characteristic emission peaks centered at 240 nm and 285 nm. Among the Co-doped hybrids, the Co_10%-_T_3_C_2_/CNT composite displayed the lowest PL intensity; this quenching effect indicates a significant suppression of photogenerated electron-hole recombination, indicating superior charge carrier separation kinetics. To further validate these findings, transient photocurrent response measurements were conducted (Figure 6b). The photocurrent density followed a parabolic trend with increasing cobalt loading, reaching its maximum for the 10 wt% Co sample. This enhancement is attributed to the synergistic interfacial coupling between the Ti_3_C_2_ MXene and the in situ grown CNTs, combined with the presence of multivalent Co active sites that facilitate both the spatial separation and rapid migration of photogenerated carriers.

Electrochemical impedance spectroscopy (EIS) was further utilized to probe the charge transfer resistance (R_ct_) at the electrode/electrolyte interface. As illustrated in the Nyquist plots (Figure 6c), the Co_10%_-Ti_3_C_2_/CNTs sample exhibits the smallest semicircle radius among all tested materials. This reduction in R_ct_ indicates significantly accelerated interfacial charge transfer and enhanced electrical conductivity, which is consistent with the observed PL quenching and high photocurrent response. Collectively, these results confirm that the 10 wt% Co loading provides an optimal configuration for minimizing charge recombination and maximizing the utilization of photogenerated carriers for photocatalytic degradation.

### 2.7. Photocatalytic Degradation Study

**Figure 7:**
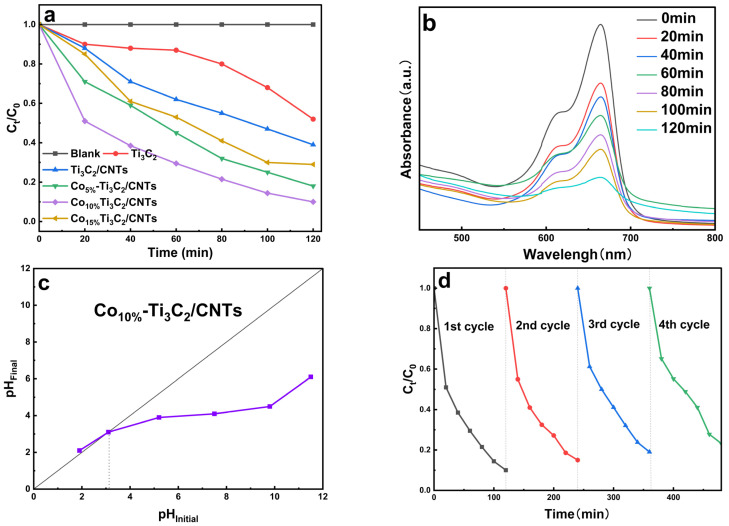
(**a**) MB degradation curves by Ti_3_C_2_, Ti_3_C_2_/CNTs, and Co-Ti_3_C_2_/CNT composites; (**b**) UV-Vis absorption spectra of MB degradation by the Co_10%_-Ti_3_C_2_/CNT composite at different time intervals; (**c**) pHpzc of the Co_10%_-Ti_3_C_2_/CNT composite; (**d**) reusability and stability test of the Co_10%_-Ti_3_C_2_/CNT composite.

Using MB as a model pollutant, the photocatalytic efficiencies of the synthesized catalysts were evaluated through the degradation of MB under visible-light irradiation (Figure 7a). Control experiments in the absence of a catalyst showed negligible MB degradation, confirming the high photostability of the dye molecules. Ti_3_C_2_ and Ti_3_C_2_/CNTs achieved degradation efficiencies of 48.2% and 61.3%, respectively, within 120 min. In contrast, the Co_10%_-Ti_3_C_2_/CNT composite exhibited superior performance, reaching a degradation rate of 90.3%. This efficiency is highly competitive with recently reported MXene-based photocatalysts (Table 2). The observed performance trend suggests that at low loading, an insufficient CNT network fails to establish adequate conductive pathways, while an increased excess of Co induces a shielding effect, where the metal species compete for incident photons and shielding of active surface sites. Thus, a 10 wt% Co loading represents an optimal threshold that maximizes the synergistic interplay between the Schottky barrier and spatial charge separation.

The time-dependent UV-vis absorption spectra (Figure 7b) illustrate a rapid and continuous decrease in the characteristic MB absorbance peak, illustrating efficient mineralization. Furthermore, the point of zero charge (pH_pzc_) for the Co_10%_-Ti_3_C_2_/CNTs was determined to be 3.12 (Figure 7c) using the pH drift method [46]. This indicates that across a broad pH range (pH > 3.12), the catalyst surface is negatively charged. This surface state significantly improves the electrostatic adsorption of cationic MB molecules, a critical precursor to efficient surface-mediated photocatalysis.

The stability and reusability of the Co_10%_-Ti_3_C_2_/CNT photocatalyst were assessed over four successive cycles (Figure 7d). The composite maintained robust activity, with a marginal 14.8% decrease in efficiency by the final cycle. This minor decline can be attributed to the irreversible adsorption of intermediate degradation products on the active sites or the inevitable loss of trace amounts of catalyst during the recovery and washing steps, rather than a fundamental degradation of the material’s structural integrity.

**Table 2:** Comparison of photocatalytic performance.

Photocatalysts	Pollutants	Light Source	Degradation Ratio (%)	Performance Improvement	Ref.
HGO@Ti_3_C_2_	MB	Visible Light	99%	1.65	[47]
ZnO/NiWO_4_/V_2_C	MB	Visible Light	91.7%	1.45	[48]
Ti_3_C_2_@UiO66	MB	Visible Light	78%	1.48	[49]
TiO_2_/CNTs	MB	Visible Light	85%	1.81	[50]
Ag/Ag_3_PO_4_/Ti_3_C_2_	CV	Visible Light	83.64%	1.7	[51]
ML-Ti_3_C_2_(OH)_2_	MB	Visible Light	81.2%	1.62	[52]
TiO2/Ti_3_C_2_/MMT	MB	Visible Light	87.2%	1.744	[53]
Ti_3_C_2_	MB	Visible Light	47.9%	1.0	This study
Ti_3_C_2_/CNTs	MB	Visible Light	61.8%	1.36	
Co_10%_-Ti_3_C_2_/CNTs	MB	Visible Light	90.3%	2.01	

**Figure 8:**
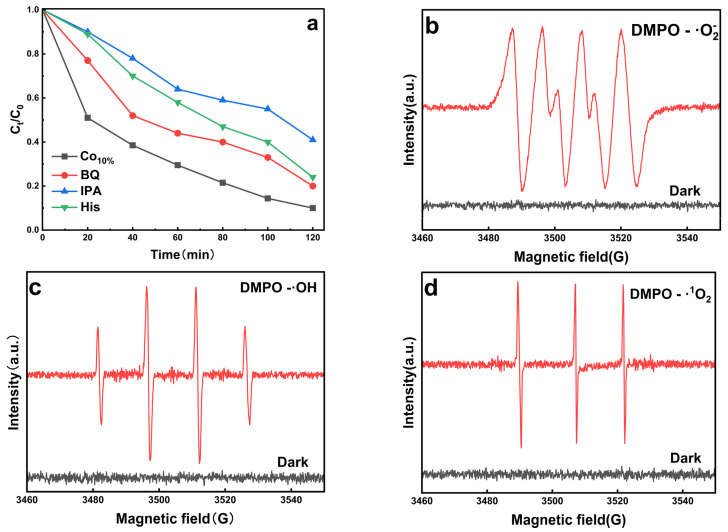
(**a**) Effects of different scavengers (BQ, IPA, and His) on the photocatalytic degradation of MB by Co_10%_-Ti_3_C_2_/CNTs; (**b**–**d**) EPR spin resonance signals of DMPO-•O_2_^−^, DMPO-•OH, and DMPO-•^1^O_2_ adducts under dark and illuminated conditions.

To determine the relative contributions of specific reactive oxygen species (ROS) in the photocatalytic degradation of MB, radical scavenging experiments were conducted (Figure 8a). Upon the addition of isopropanol (IPA), a potent hydroxyl radical (•OH) scavenger, the degradation efficiency plummeted from 90.3% to 59.2%. This pronounced inhibition identifies •OH as the primary dominant active species in the Co-Ti_3_C_2_/CNT system. The introduction of benzoquinone (BQ), a superoxide radical (•O_2_^−^) scavenger, resulted in a moderate reduction in efficiency to 79.9%, indicating that •O_2_^−^ plays a secondary role. Similarly, the addition of L-histidine (His) to quench singlet oxygen (•^1^O_2_) led to a mild decrease in activity (to 75.8%), suggesting that •^1^O_2_ also contributes as a minor active species. These results collectively demonstrate that while multiple ROS are involved, the •OH-driven oxidation pathway is the primary mechanism for MB mineralization.

Electron paramagnetic resonance (EPR) spectroscopy was further employed to provide in situ pattern identification of these ROS [54,55,56]. As illustrated in Figure 8c, a high-intensity DMPO-•OH adduct signal was detected under visible-light irradiation, characterized by the signature 1:2:2:1 peak intensity ratio. Concurrently, the four-line signal typical of DMPO-•O_2_^−^ was clearly observed (Figure 8b), confirming the reduction of adsorbed oxygen. Furthermore, a triplet signal with a 1:1:1 intensity ratio (Figure 8d) validates the generation of •^1^O_2_. The significant intensity of these signals under light, compared with their absence in the dark, perfectly aligns with the scavenging results. The unique heterojunction structure and interfacial electron-transfer pathways within the Co-Ti_3_C/CNT system ensure continuous and efficient generation of these three active species, thereby greatly accelerating the catalytic degradation process.

### 2.8. Degradation of Other Pollutants

**Figure 9:**
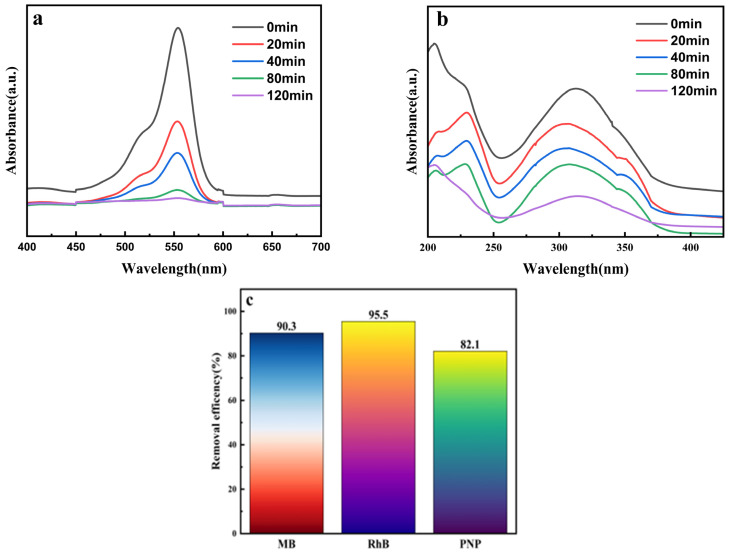
(**a**) Time-dependent UV-vis absorption spectra during the photodegradation of RhB; (**b**) time-dependent UV-vis absorption spectra during the photodegradation of PNP; (**c**) comparative degradation efficiencies of the Co_10%_-Ti_3_C_2_/CNT composite for various organic pollutants under visible-light irradiation.

Beyond its performance with MB, the Co_10%_-Ti_3_C_2_/CNT composite demonstrated robust and versatile catalytic activity toward the degradation of Rhodamine B (RhB) and p-Nitrophenol (PNP). The time-dependent UV-vis absorption spectra for the degradation of RhB and PNP are presented in Figure 9a and Figure 9b, respectively. The systematic attenuation of the characteristic absorption peaks for both pollutants confirms that the optimized heterostructure effectively facilitates the mineralization of varied organic contaminants under visible-light irradiation.

Figure 9c illustrates the degradation efficiencies of the Co_10%_-Ti_3_C_2_/CNT composite against three pollutants (MB, RhB, and PNP) under 120 min of visible light irradiation. The results indicate that the composite exhibits higher degradation efficiency for dye pollutants compared to phenolic pollutants. As organic dyes, RhB and MB typically possess strong light absorption and conjugated structures with chromophores that are more susceptible to oxidative cleavage. Consequently, they undergo more rapid degradation driven by photogenerated charge carriers. In contrast, PNP is an aromatic system containing nitro and phenolic hydroxyl groups; its mineralization often requires multistep transformations and can be influenced by variations in pH, dissolved oxygen, and specific radical species, leading to a relatively lower degradation rate within the same timeframe.

### 2.9. Photocatalytic Degradation Mechanism

**Figure 10:**
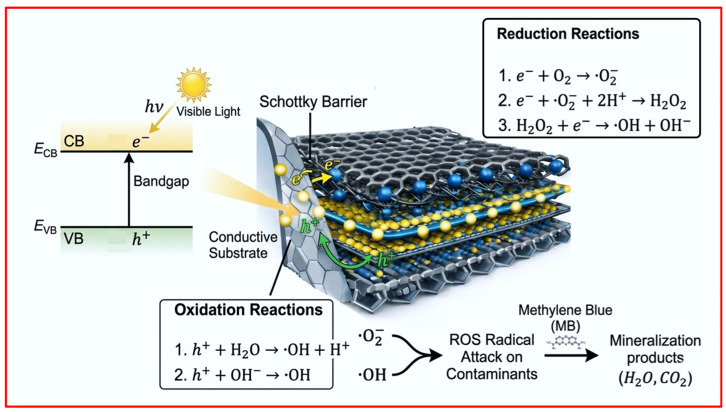
Proposed mechanism for the visible-light photocatalytic degradation of MB by the Co_10%_-Ti_3_C_2_/CNT composite.

The proposed photocatalytic mechanism for the Co-Ti_3_C_2_/CNT system under visible-light irradiation is illustrated in the schematic (Figure 10). Upon excitation, photogenerated electrons (e^−^) in the Co active species, comprising both metallic Co^0^ and oxide/hydroxide phases, are promoted from the valence band (VB) to the conduction band (CB). Due to the metallic nature and high work functions of both the Ti_3_C_2_ MXene and the in situ grown CNTs, their Fermi levels (E_f_) are positioned lower than the CB of the Co semiconductor phases. This energy gradient drives the rapid migration of photogenerated electrons from the Co species onto the Ti_3_C_2_ substrate and the interconnected CNT network.

The high-density CNTs on the MXene surface function as a 3D electron transport “highway,” facilitating the rapid dispersion and accumulation of electrons across the composite surface. This interfacial charge redistribution induces band bending within the Co species, resulting in the formation of a Schottky barrier at the semiconductor–metal (Co-Ti_3_C_2_/CNTs) interface. This barrier acts as a directional electron trap, effectively preventing the backflow of carriers to the Co active sites and significantly suppressing the recombination of photogenerated e^−^/h^+^ pairs. On the conductive surfaces of the CNTs and Ti_3_C_2_, the trapped electrons react with dissolved oxygen to form superoxide radicals (•O_2_^−^), which subsequently undergo multistep reductions to generate highly oxidative hydroxyl radicals (•OH). Simultaneously, the photogenerated holes (h^+^) remaining in the VB of the Co species react with surface-adsorbed H_2_O or OH^−^ to produce additional •OH. These potent reactive species, primarily •OH and secondary •O_2_^−^, collectively mineralize MB molecules into H_2_O, CO_2_, and other colorless intermediates.

## 3. Experimental Section

### 3.1. Materials

Ti_3_AlC_2_ powder (purity > 99.9%) was purchased from Nanjing Jicang Nano Technology Co., Ltd. (Nanjing, China) Hydrochloric acid (HCl, 37%), lithium fluoride (LiF, purity > 99%), anhydrous ethanol (purity > 99.9%), methylene blue (MB, purity > 98.5%), cobalt (II) acetylacetonate (purity > 98%), and cobalt (II) nitrate hexahydrate (purity > 99.99%) were all acquired from Shanghai Aladdin Biochemical Technology Co., Ltd. (Shanghai, China). Short-cut carbon fiber (carbon content > 97%) was purchased from Carbonene Technology Co., Ltd. (Shenzhen, China).

### 3.2. Preparation of Ti_3_C_2_

Few-layer Ti_3_C_2_ nanosheets were prepared via in situ etching using LiF/HCl. Typically, 1 g of LiF powder was slowly dissolved in 20 mL of 9 M hydrochloric acid under continuous stirring until a clear solution was obtained. Subsequently, 1 g of Ti_3_AlC_2_ powder was added in batches to the acidic solution. The mixture was maintained at 45 °C for 24 h under magnetic stirring to ensure the complete removal of the aluminum (Al) layers. The resulting suspension was subjected to repeated cycles of centrifugation and washing with deionized water until the supernatant reached a near-neutral pH. Following this, the product underwent probe ultrasonication to facilitate exfoliation, after which the supernatant was collected and freeze-dried to yield Ti_3_C_2_ powder.

### 3.3. Preparation of Co-Ti_3_C_2_/CNT Composite

The Co-Ti_3_C_2_/CNT composites were synthesized through a multistep process involving microwave-assisted growth and subsequent hydrothermal doping. Initially, equal masses (100 mg) of Ti_3_C_2_ MXene and cobalt (II) acetylacetonate were placed in a mortar with 5 mL of anhydrous ethanol and ground for 5 min. After drying, the mixture was placed in an alumina crucible and covered with approximately 5 mg of chopped carbon fiber. The sample was then subjected to microwave irradiation at 800 W for 30 s. This process was repeated: the resulting black intermediate was re-mixed with an equivalent weight of cobalt (II) acetylacetonate, ground with ethanol, dried, and irradiated for an additional 30 s to ensure the robust formation of the Ti_3_C_2_/CNT hybrid structure.

**Figure 11:**
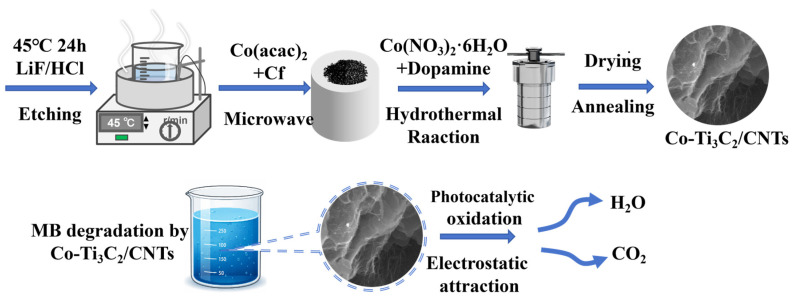
Schematic of the synthesis of the Co-Ti_3_C_2_/CNT composite.

Subsequently, 100 mg of the Ti_3_C_2_/CNT powder was ultrasonically dispersed in 20 mL of anhydrous ethanol, followed by the addition of 5 mg of dopamine to facilitate interfacial bonding. Cobalt (II) nitrate hexahydrate was then introduced at varying mass ratios (5 wt%, 10 wt%, and 15 wt%). After stirring at room temperature for 1 h, the suspension was transferred to a Teflon-lined stainless-steel autoclave and maintained at 120 °C for 6 h. The product was collected via centrifugation, washed thoroughly, and freeze-dried. Finally, the obtained powder was annealed in a tube furnace at 300 °C for 3 h under an inert atmosphere to yield the final Co-Ti_3_C_2_/CNT photocatalysts, as shown in schematic Figure 11.

### 3.4. Characterization

The phase composition was analyzed using X-ray diffraction (XRD, D8 Advance, Bruker, Karlsruhe, Germany). The material morphology and interface were observed using scanning electron microscopy (SEM, FEI Inspect F50, Thermo Fisher Scientific, Waltham, MA, USA) and transmission electron microscopy (TEM, JEM-2100F, JEOL, Tokyo, Japan). X-ray photoelectron spectroscopy (XPS, Thermo Fisher Scientific, Waltham, MA, USA) was utilized to explore the elemental valence states and surface chemical bonding. Fourier transform infrared (FT-IR) spectra were obtained using a spectrometer (IR Prestige-21, Shimadzu, Kyoto, Japan). Ultraviolet-visible spectroscopy (UV-vis, JASCO V-600, Jasco, Tokyo, Japan) was used to study the light absorption characteristics. The specific surface area was determined by analyzing N_2_ adsorption–desorption isotherms using a Brunauer–Emmett–Teller (BET, ASAP2460, Micromeritics, Norcross, GA, USA) apparatus. Electron paramagnetic resonance (EPR, A300, Bruker, Billerica, MA, USA) spectroscopy was performed at room temperature to evaluate free radical generation. Photoelectrochemical tests were performed using a 0.5 M Na_2_SO_4_ solution as the electrolyte and a 300 W xenon lamp as the light source.

### 3.5. Photocatalytic Degradation Analysis

Photocatalytic performance was evaluated using MB as a model organic pollutant. Typically, 60 mg of the photocatalyst was dispersed in 50 mL of an aqueous MB solution (60 mg/L). Prior to irradiation, the suspension was magnetically stirred in the dark for 40 min to obtain adsorption–desorption equilibrium. Photocatalytic degradation was then initiated using a 300 W Xenon lamp (CEL-PF300-T8, AuLight, Beijing, China) equipped with a 420 nm cutoff filter to ensure visible-light irradiation. At 20 min intervals, 3 mL aliquots were withdrawn and centrifuged (7000 g, 5 min) to remove the catalyst particles. The residual MB concentration was monitored via UV-vis spectrophotometry (JASCO V-600) across the 200–800 nm wavelength range. Moreover, the recyclability and stability of the Co-Ti_3_C_2_/CNT composite were investigated. After each cycle, the used Co-Ti_3_C_2_/CNT catalyst was collected via centrifugation, washed, and dried for subsequent use.

To elucidate the primary reactive oxygen species (ROS) involved in the degradation process, radical scavenging experiments were conducted. Histidine (His), p-benzoquinone (BQ), and isopropanol (IPA) were employed as scavengers for singlet oxygen (^1^O_2_), superoxide radicals (•O_2_^−^), and hydroxyl radicals •OH), respectively. The experimental conditions for scavenging tests were kept identical to those of the standard photocatalytic runs.

The point of zero charge (pH_pzc_) was measured using the pH drift method. Briefly, 0.5 g of the composite was transferred to 25 mL of a 0.01 M NaCl solution, adjusted to different initial pH values (2~12). After standing for 24 h, the final pH was measured. The pH_pzc_ was recorded from the intersection point of the initial and final pH values.

## 4. Conclusions

In this work, a 3D hierarchical Co-Ti_3_C_2_/CNT composite was successfully fabricated through a synergistic combination of microwave-assisted in situ growth and a facile hydrothermal process. The resulting structure effectively addresses the primary limitations of 2D MXenes by utilizing CNTs as structural spacers to prevent nanosheet restacking and as conductive highways to accelerate electron transport. Characterization results demonstrate that the optimized Co_10%_-Ti_3_C_2_/CNT hybrid possesses a high specific surface area and significantly broadened visible-light absorption. Notably, this composite achieved a superior photocatalytic degradation rate of 90.3% for MB within 120 min, outperforming pristine Ti_3_C_2_ and several recently reported MXene-based composites. This enhanced performance is attributed to the formation of a robust interfacial Schottky junction, which acts as a directional electron trap to suppress charge recombination. Furthermore, the material exhibited excellent structural stability and reusability over multiple cycles. Given its rapid, cost-effective synthesis and high catalytic efficiency, this work provides a versatile strategy for the development of advanced MXene-based heterostructures for sustainable environmental remediation.

## Figures and Tables

**Table 1:** Specific surface area (S_BET_), pore volume (V_pore_), and average pore diameter (D_pore_) of the composites.

Samples	S_BET_ (m^2^/g)	V_pore_ (cm^3^/g)	D_pore_ (nm)
Co_5%_	33.1902	0.152258	18.3498
Co_10%_	39.4531	0.215512	21.8500
Co_15%_	32.5727	0.186772	22.9360

## Data Availability

The original contributions presented in this study are included in the article. Further inquiries can be directed to the corresponding author(s).
